# Rheumatoid Arthritis is an Autoimmune Disease Triggered by *Proteus* Urinary Tract Infection 

**DOI:** 10.1080/17402520600576578

**Published:** 2006-03

**Authors:** Alan Ebringer, Taha Rashid

**Affiliations:** School of Biomedical and Health Sciences Kings College London London UK

## Abstract

Rheumatoid arthritis (RA) is a chronic and disabling polyarthritic disease, which affects mainly women in middle and old age.

Extensive evidence based on the results of various microbial, immunological and molecular studies from different parts of the world, shows that a strong link exists between *Proteus mirabilis* microbes and RA. We propose that sub-clinical *Proteus* urinary tract infections are the main triggering factors and that the presence of molecular mimicry and cross-reactivity between these bacteria and RA-targeted tissue antigens assists in the perpetuation of the disease process through production of cytopathic auto-antibodies.

Patients with RA especially during the early stages of the disease could benefit from *Proteus* anti-bacterial measures involving the use of antibiotics, vegetarian diets and high intake of water and fruit juices such as cranberry juice in addition to the currently employed treatments.


*Clinical & Developmental Immunology*, March 2006; 13(1): 41–48

### Rheumatoid arthritis is an autoimmune disease triggered by *Proteus* urinary tract infection

ALAN EBRINGER & TAHA RASHID

*School of Biomedical and Health Sciences, Kings College London, London, UK*

**Abstract**

Rheumatoid arthritis (RA) is a chronic and disabling polyarthritic disease, which affects mainly women in middle and old age.

Extensive evidence based on the results of various microbial, immunological and molecular studies from different parts of the world, shows that a strong link exists between *Proteus mirabilis* microbes and RA. We propose that sub-clinical *Proteus* urinary tract infections are the main triggering factors and that the presence of molecular mimicry and cross-reactivity between these bacteria and RA-targeted tissue antigens assists in the perpetuation of the disease process through production of cytopathic auto-antibodies.

Patients with RA especially during the early stages of the disease could benefit from *Proteus* anti-bacterial measures involving the use of antibiotics, vegetarian diets and high intake of water and fruit juices such as cranberry juice in addition to the currently employed treatments.

**Keywords:** *Humoral autoimmunity,* Proteus mirabilis, *rheumatoid arthritis, urinary tract infection*

### Introduction

Rheumatoid arthritis (RA) is a chronic inflammatory joint disease, which affects millions of people all around the world, with a prevalence rate ranging from 0.5 to 1% (Lawrence et al. 1998). The disease in the majority of patients takes a mild to moderate course, whilst in others it has a more disabling consequence, which might have a great effect on the socio-economic status of the patient (Cooper 2000).

RA affects individuals of middle age groups and occurs three times more frequently in women than in men.

### Aetiopathogenesis

A general scientific consensus exists, which considers RA as an immune-mediated disease that could possibly be triggered by an environmental (microbial) factor in a genetically susceptible individual.

Extensive evidence supports the role of cellular and humoral autoimmunity in the development of RA, and some of these are listed as follows:

1. Predominant role of B lymphocytes in the pathogenesis of RA (Weyand et al. 2005) and signs of accumulations of immunoglobulins and other inflammatory products such as complements at the site of synovial pathological lesions in RA patients (Low and Moore 2005).
2. Detection of elevated levels of auto-antibodies in the serum and/or synovial fluid of patients with RA (Rantapaa-Dahlqvist 2005).
3. Significant improvements in RA disease parameters following B cell depletion therapy, e.g. with the use of anti-CD20 antibodies (Perosa et al. 2005).

### *Role of HLA genes in RA*

The role of genetics in development of RA has been examined mainly through family, twin and molecular analytical studies. For instance, familial distribution of RA among first-degree relatives (Deighton et al. 1992a) and twins (MacGregor et al. 2000), indicates that RA runs in some families, basically supporting the

Correspondence: A. Ebringer, Infection and Immunity Group, School of Biomedical and Health Science, King's College London, 150 Stamford Street, London SE1 9NN, UK. Tel: 44 20 7848 4302. Fax: 44 20 7848 4500. E-mail: alan.ebringer@kcl.ac.uk

ISSN 1740-2522 print/ISSN 1740-2530 online © 2006 Taylor & Francis
DOI: 10.1080/17402520600576578

concept of a genetic contribution, but at the same time arguing against the proposition that RA could be considered as a purely genetic disorder. However, in two separate genome-wide screenings with affected sib pairs, it has been shown that HLA haplotypes had the largest genetic contribution in RA (Cornelis et al. 1998; Jawaheer et al. 2001).

Nearly three decades ago, HLA-DR4 was the first genetic marker among other class II molecules, found to be significantly liked with RA susceptibility (Stastny 1976). It has been shown later that various other HLA-DRB1 alleles were either associated (HLA-DRB*0101, *0102, *0401, *0404, *0405, *1001 and 1402) or not associated (HLA-DRB*0103, *0402, *0403 and *0408) with RA (Newton et al. 2004), based on certain differences occurring within amino acid sequences at the location 69–74 "EQRRAA" which has been termed as the "shared epitope" (SE) moiety (Gregersen et al. 1987).

Regardless of the difference in the distribution of HLA genes among various ethnic groups, it has been reported that more than 95% of patients possess at least one of the RA-linked HLA-DR molecules (Weyand et al. 1992), which contain the "SE" amino acid sequence. Further to its role in conferring the disease susceptibility, these RA-associated HLA-DRB1 alleles have also been reported to have a great impact on the disease severity and extra-articular manifestations in patients with RA (Turesson et al. 2005).

### *Immunological and molecular links between RA and* Proteus

During the last 3 decades an extensive literature on the link between *Proteus* microbes and RA has been published in peer-reviewed journals. These studies were carried out by various independent groups worldwide. Some of this evidence, listed in chronological orders, can be summarized as follows: (Table I)

1. In early 1970s, a group from Tennessee reported an increase in the geometric mean titres of antibodies to *Proteus* OXK and herpes virus hominis but not to 28 other infectious disease antigens in RA patients when compared to controls (Chandler et al. 1971).
2. In mid 1980s, our group reported for the first time that antibody levels against *P. mirabilis*, a urinary pathogen, were significantly elevated in RA patients when compared to those with ankylosing spondylitis (AS) or healthy control subjects (Ebringer et al. 1985).
3. In early 1990s, we have identified an amino acid sequence homology between the "EQ/KRRAA" amino acid motif present in RA HLA-susceptibility molecules and the "ESRRAL" sequence present in *P. mirabilis* haemolysins (Ebringer et al. 1992).
4. In mid 1990s, our group has found another molecular homology between "LRREI" amino acid sequence present in type XI collagen and "IRRET" amino acid motif present in *Proteus* urease enzymes (Wilson et al. 1995).
5. In late 1990s, *in vitro* and *in vivo* immunological cross reactivities have been observed between synthetic peptides from *Proteus* haemolysin and urease enzyme molecules and those of HLA-DR1/DR4 and collagen self-antigens (Tiwana et al. 1999). Furthermore, Senior and colleagues from Dundee, have found that *Proteus mirabilis* is the most prominent microbe encountered in the urine of RA patients, who had bacteriuria more frequently than age- and sex-matched healthy controls (Senior et al. 1999).
6. In the year 2003, our group has shown that anti-ESRRAL *Proteus* antibodies from patients with active RA can haemolyse sheep red blood cells (SRBC) coated with HLA-DR4/1-containing "EQRRAA" but not HLA-B27-containing "QTDRED" amino acid synthetic peptides more frequently than patients with AS and healthy controls. By contrast, active AS patients with high anti-QTDRED *Klebsiella* antibodies had higher levels of an *in vitro* haemolytic reactions on SRBCs coated with "QTDRED" but not "ESRRAL" synthetic peptides when compared to patients with RA or to healthy controls (Wilson et al. 2003). Hence, RA and AS groups of patients could behave as reciprocal controls to each other concerning the involvement of antibodies against *Proteus* antigens in RA and *Klebsiella* antigens in AS. Blood donors did not react against either *Proteus* or *Klebsiella* antigens.
7. Between the year 1985 and 2003, many serological studies have been carried out by various independent groups. Cumulatively, they show that elevated antibodies against various antigens from *Proteus mirabilis* were observed in more than 1350 RA patients from 15 different countries worldwide when compared to corresponding healthy controls (Ebringer et al. 2003).
8. In the year 2004, a collaborative study was carried out involving Finish and Japanese patients with RA. It was observed that these patients were all showing significantly elevated levels of IgG antibodies to whole and synthetic peptide antigen preparations from *P. mirabilis* but not to those from *E. coli* and *S. marcescens* bacteria when compared to corresponding healthy controls or patients with systemic lupus erythematosus (Rashid et al. 2004).
9. In a recent multi-centre prospective study, it has been shown that rheumatoid factor-(RF) positive patients with RA had significantly elevated levels of IgM and IgA antibodies against *Proteus mirabilis* and IgA antibodies against *E. coli* when compared to disease and healthy control subjects

Table I. Chronologically listed evidence of microbiological, molecular and immunological links between *Proteus* microbes and rheumatoid arthritis

| Reference | Basic results | Methods used |
|---|---|---|
| Chandler et al. (1971) | Elevated antibodies to *Proteus* OXK and herpes virus but not to 22 other microbial agents in RA patients when compared HCs. | Direct agglutination assay |
| Ebringer et al. (1985) | Elevated antibodies to *P. mirabilis* but not *Klebsiella* microbes in patients with RA when compared to AS patients and HCs. | Indirect agglutination assay |
| Abuljadayel et al. (1988) | Elevated anti-*Proteus* antibodies in RA patients compared to HCs. | IIF |
| Rogers et al. (1988) | Elevated levels of anti-*Proteus* antibodies in RA patients compared to other disease or healthy controls. | ELISA |
| Nasonova et al. (1989) | Increased anti-*Proteus* antibodies in the majority of Russian RA patients when compared to corresponding disease or healthy controls. | ELISA |
| Wilson et al. (1990) | Increased isolation of *P. mirabilis* from urine of RA patients when compared to patients with OA or to HCs. | CLED (Oxoid Ltd) growth and API20E (bioMerieux, Inc) identification methods |
| Deighton et al. (1992b) | Absence of positive correlations between elevated antibodies to *P. mirabilis* and antibodies against 4 viruses and 8 auto-antigens in patients with RA when compared to HCs | IIF |
| Ebringer et al. (1992) | Molecular homology between *Proteus* haemolysins and HLA-DR4/1 antigens. | SwissProt and GenBank database search |
| Ebringer et al. (1993) | Increased isolation rate of *P. mirabilis* from urine of female more than male patients with RA and in RA patients as a whole when compared to healthy subjects. | CLED growth and API20E (bioMerieux, Inc) identification methods |
| Fielder et al. (1995) | Elevated antibodies to *P. mirabilis* but not to *E. coli* or *S. Typhi* bacteria in French patients with RA when compared to corresponding HCs. | ELISA and IIF |
| Subair et al. (1995) | Elevated antibodies to *P. mirabilis* but not to *E. coli* or other normal bowel microbial inhabitants in Bermudian patients with RA when compared to corresponding HCs. | ELISA and IIF |
| Wilson et al. (1995) | Molecular similarities between *Proteus* urease and collagen type XI antigens. Elevated antibodies to *Proteus* haemolysin and urease as well as to HLA-DR4/1 and collagen antigenic peptides. | SwissProt and GenBank database search and ELISA |
| Tiwana et al. (1996) | Elevated antibodies to *Proteus* but not to *Serratia*, *Escherichia* and *Pseudomonas* bacteria in RA patients when compared to HCs. | ELISA |
| Wanchu et al. (1997) | Elevated antibodies to *P. mirabilis* but not to *S. typhi* in Indian patients with RA when compared to OA patients and to HCs. | Tube agglutination |
| Wilson et al. (1997) | A positive correlation between high serum anti-*Proteus* antibody levels and urine *Proteus* isolation rates in patients with RA. | ELISA and API20E methods |
| Blankenberg-Sprenkels et al. (1998) | Elevated anti-*Proteus* but not anti-*Klebsiella* antibodies in Dutch RA patients when compared to patients with AS or to HCs. | IIF |
| Tiwana et al. (1999) | In vitro and in vivo immunological cross-reactivities between antibodies against *P. mirabilis* and self-antigenic peptide molecules. | ELISA and peptide binding dilution assay |
| Rashid et al. (1999) | Elevated antibodies to *P. mirabilis* in Spanish and Norwegian patients with RA when compared to corresponding HCs. | IIF |

Table I – *continued*

| Reference | Basic results | Methods used |
|---|---|---|
| Senior et al. (1999) | Sub clinical *Proteus* bacteriuria and elevated levels of anti-*Proteus* antibodies in urine and serum of patients with RA when compared to HCs. | ELISA and Immunoblot |
| Ushakova et al. (2000) | Elevated IgM and IgG antibodies to *P. mirabilis* in Russian patients with RA when compared to HCs. | ELISA |
| Wilson et al. (2003) | In vitro immunological cytotoxicity reaction occurring between anti-*Proteus* and anti-HLA-DRB1 antibodies with the corresponding crossreactive peptide molecules. | ELISA and sheep red cell haemolytic assays |
| Rashid et al. (2004) | Finish and Japanese patients with RA had elevated antibodies against whole and synthetic peptide antigenic preparations from *P. mirabilis* but not those from *E. coli* and *S. marcescens* when compared to corresponding HCs. | ELISA and IIF |
| Newkirk et al. (2005) | Elevated IgM and IgA antibodies to *P. mirabilis* and IgA antibodies to *E. coli* but not to 6 other bacterial and viral agents in rheumatoid factor positive RA patients when compared to those with other rheumatic diseases. | ELISA |

ELISA = Enzyme linked immunosorbent assay; HCs = Healthy controls; IIF = Indirect immunofluorescence; RA = Rheumatoid arthritis; AS = Ankylosing spondylitis; OA = Osteoarthritis; CLED = Cystine lactose-electrolyte deficient.

(Newkirk et al. 2005). However, many other studies carried out previously had shown that only anti-*Proteus* but no anti-*E. coli* antibodies were observed to be elevated in RA patients (Fielder et al. 1995, Subair et al. 1995; Tiwana et al. 1996; Tani et al. 1997; Rashid et al. 2004).

10. In another study carried out by a group from Los Angeles, RA patients were reported to have IgA anti-*Proteus* antibodies detectable more than in healthy controls. However, several molecules from this microbe had shown reduced IgA immune responses compared to controls (Weisbart et al. 2005). It has been suggested that the occurrence of some "holes" in the IgA immune repertoire for certain antigens in *Proteus* bacteria could explain the possibility that RA patients are more liable to harbour this microbe and to have more frequent bouts of (albeit, sub-clinical) infections, with a consequent intermittent and/or progressive enhancement of anti-*Proteus* antibody responses.
11. Most recently, our group has studied the humoral immune responses against a new antigenic moiety from *Proteus* urease F enzyme, which was found to be non homologous to RA-targeted synovial tissue structures. We have shown that active RA patients had significantly elevated IgG and IgM anti-*Proteus* peptide antibodies when compared to healthy controls (Rashid et al. submitted). This latter finding argues against the suggestion that elevated antibodies against the *Proteus* crossreactive antigens in RA patients could be an epiphenomenona resulting from increased exposure of auto-antigens due to pathological damages produced by bacterial triggers.

### Proteus *urinary tract infections and RA*

Various microbiological and immunological data results support the suggestion that there is a link between RA and urinary tract infections (UTI) mainly caused by *Proteus* (Table II).

Patients with RA were reported to have a higher frequency of recurrent UTIs (Lawson and Maclean 1966; Tishler et al. 1992). Urine samples from RA patients yield higher isolation rates of *P. mirabilis* microbes than patients with osteoarthritis or healthy controls (Ebringer et al. 1993). RA patients had higher levels of antibodies against *Proteus* in their urine when compared to those of healthy subjects (Senior et al. 1999) and significant correlation was detected between serum anti-*Proteus* antibodies and *Proteus* urinary isolation rates in RA patients when compared to healthy controls (Wilson et al. 1997). Furthermore, RA patients group who were receiving a vegetarian diet had a drop in the levels of antibodies against *P. mirabilis* but not *E. coli*, when compared to those patients who were omnivores (Kjeldsen-Kragh et al. 1995). This dietary treatment may have exerted its specific anti-microbial effect via the actions of lignans and phytoestrogen metabolites (Adlercreutz et al.

Table II. Direct and indirect evidence of the links between *Proteus* UTIs and RA

| |
|---|
| *Direct evidence:* |
| 1) *Proteus mirabilis* bacteria were isolated more frequently in patients with RA than those with other disease and healthy controls (Wilson et al. 1990; Senior et al. 1999). |
| 2) A positive correlation has been detected between anti-*Proteus* antibodies and the rate of isolation of these microbes from urine of RA patients when compared to healthy subjects (Wilson et al. 1997). |
| 3) RA patients receiving vegetarian diet are more liable to have decreased levels of anti-*Proteus* antibodies than omnivores (Kjeldsen-Kragh et al. 1995). |
| 4) Elevated levels of anti-*Proteus* antibodies were observed in sera of RA patients coming from 15 different countries (Ebringer et al. 2003). |
| *Indirect evidence:* |
| 1) Patients with severe RA had a higher frequency of recurrent UTIs (Tishler et al. 1992). |
| 2) Women are more liable to develop recurrent UTIs (Franco 2005). This finding in combination to point number 1 above, could explain why RA is more common among women than men. |
| 3) *Proteus* bacteria, which are urease-positive, account for 15% of UTIs, and affect the upper urinary tract, especially kidneys, whilst *E. coli*, another urogenic but urease-negative bacteria, account for the majority of UTIs and mainly involve bladder (Cattell 2005). |
| 4) Patients with recurrent UTIs are more likely to respond to high daily intake of cranberry juice (Raz et al. 2004). Whether cranberry juice could have a beneficial effect in patients with RA needs to be investigated in a prospective controlled study. |

1986), which are known to possess anti-bacterial activity *in vitro* (Ito et al. 1982).

Females are more vulnerable to develop UTIs than males and up to 60% of women have been reported to develop at least one episode of UTI in their lifetime (Foxman et al. 2000). A significant number of these women might have recurrent infections, and the chance of infection by *Proteus* species was found to increase with age (Nicolle 1996).

The increased incidence of UTIs in RA patients and the more frequent occurrence of UTIs in females, especially those of middle age and elderly groups might give an answer to the higher prevalence of RA among women.

### Molecular mimicry as a plausible aetiopathogenetic mechanism

Molecular and immunological inter-relation between the triggering aetiological factor, namely *Proteus mirabilis* antigens, and targeted synovial tissue structures expressing HLA molecules that contain "SE" amino acid motif and collagen type XI, in RA could be explained by the molecular mimicry or cross-reactivity mechanism (Ebringer et al. 2005). High antibody titres against *Proteus* haemolysin and urease antigens in patients with active RA could bind to cross-reactive self antigens and consequently result in production of various inflammatory and immunological damaging mediators based on the mechanism of antibody-mediated cytotoxicity reactions (Figure 1). Damage to the synovial and other joint structures could result in the release of self-antigenic particles and auto-antibody production. Anti-*Proteus* cross-reactive antibodies, which might result from recurrent, albeit subclinical UTIs, together with high levels of secondary auto-antibodies will bind to RA-targeted antigens in the synovial tissues. These immunological reactions could coincide with the initiation of clinical and laboratory exacerbations and further pathological damages.

### Proposal of a new therapeutic strategy

Currently patients with RA are treated with various therapeutic strategies involving the use of anti-inflammatory, immunosuppressive and biological agents as well as non-medical treatment modalities (Genovese and Harris 2005).

Although, the current medical treatments, especially those involving anti-tumour necrosis factor therapies (Haraoui 2005), have a promising beneficial effect particularly in easing or even halting the disease progress, they are expensive (Merkesdal et al. 2004) and cause side-effects. In order, to achieve a prolonged clinical remission, continuous immunosuppression with these drugs is required because this kind of treatment could not eradicate the causative microbial agents.

Based on the existing evidence for involving *Proteus* bacteria in the pathogenesis of RA, it is logical to propose a new therapeutic modality in the management of RA, which could be implemented in conjunction with other currently used treatments. This new treatment includes anti-*Proteus* measures involving the use of *Proteus*-sensitive antibiotics with dietary manipulations in the forms of vegetarian diet (Kjeldsen-Kragh et al. 1995) and high daily intake of water and fruit juices containing fructose such as cranberry juice (Rashid et al. 2001).

It could also be possible to make a vaccine mainly derived from *Proteus* antigenic molecules that do not contain the cross-reactive epitopes, in order to prevent susceptible individuals of acquiring *Proteus* UTIs and decrease the chance of developing RA or at least limit further damages in those with established disease.

### Conclusion

Based on the results of various studies carried out in relation to *Proteus* microbes, it could be said that compelling evidence exists linking this microbe to RA, starting with recurrent sub-clinical *Proteus* UTIs and ending in the full development of RA. To prove the scientific logic of this possibility, and its benefit to patients clinical trials using anti-*Proteus* measures in RA are required to be carried out in prospective longitudinal studies.

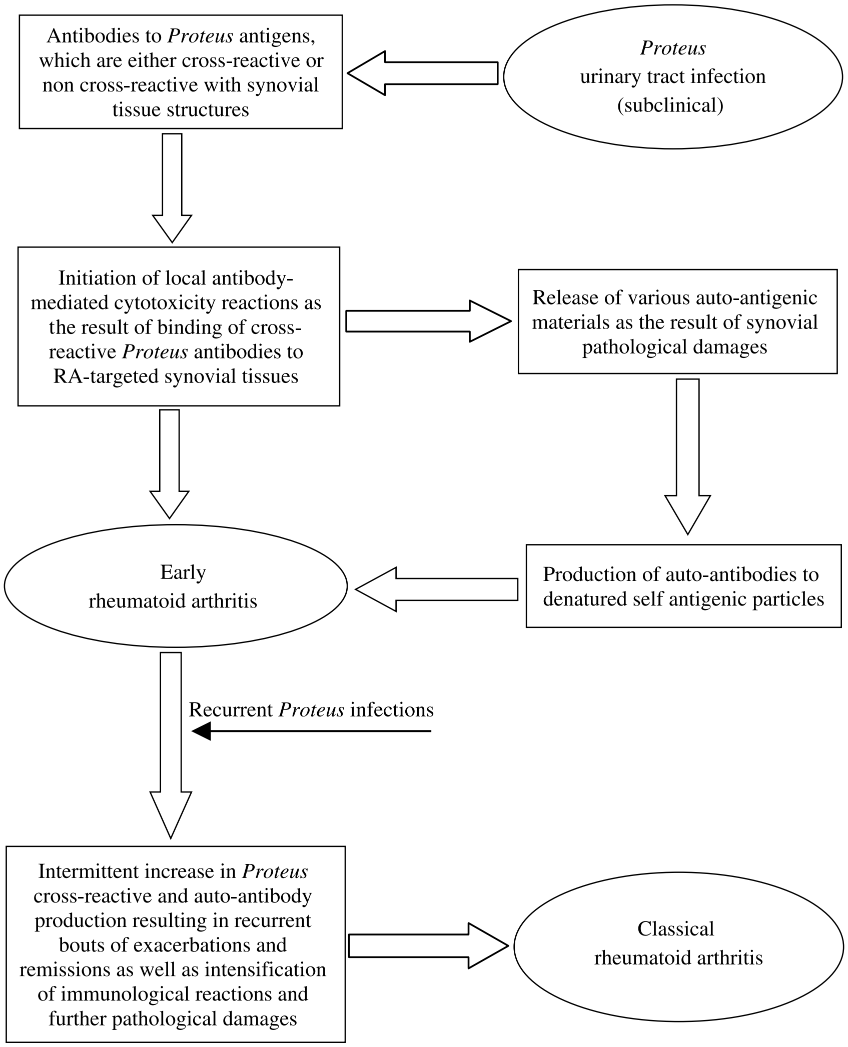

Figure 1. Schematic representation of pathological steps in the development of rheumatoid arthritis.

### Acknowledgements

We thank the Trustees of the Middlesex Hospital, the Arthritis Research Campaign (Grant EO514) and "American Friends of King's College London" for their support.

### References

Abuljadayel I, Cox N, Ebringer A. 1988. Antibodies to *Proteus* in rheumatoid arthritis (RA) and to *Klebsiella* in ankylosing spondylitis (AS) measured by immunofluorescence. Br J Rheumatol 27(Suppl. 2): Abst. 2.

Adlercreutz H, Fotsis T, Bannwart C, Wahala K, Makela T, Brunow G, Hase T. 1986. Determination of urinary lignans and phytoestrogen metabolites, potential antiestrogens and anticarcinogens, in urine of women on various habitual diets. J Steroid Biochem 25:791–797.

Blankenberg-Sprenkels SH, Fielder M, Feltkamp TE, Tiwana H, Wilson C, Ebringer A. 1998. Antibodies to *Klebsiella pneumoniae* in Dutch patients with ankylosing spondylitis and acute anterior uveitis and to *Proteus mirabilis* in rheumatoid arthritis. J Rheumatol 25:743–747.

Cattell WR. 2005. Lower and upper urinary tract infections in the adult. In: Davison AM, Cameron JS, Grunfeld J, Ponticelli C, Ritz E, Winearls CG, van Ypersele C, editors. Oxford textbook of clinical nephrology. Oxford: Oxford University Press, 1111–1129.

Chandler RW, Robinson H, Masi AT. 1971. Serological investigations for evidence of an infectious aetiology of rheumatoid arthritis. Ann Rheum Dis 30:274–278.

Cooper NJ. 2000. Economic burden of rheumatoid arthritis: A systematic review. Rheumatology 39:28–33.

Cornelis F, Faure S, Martinez M, Prud'homme JF, Fritz P, Dib C, Alves H, Barrera P, de Vries N, Balsa A, et al. 1998. New susceptibility locus for rheumatoid arthritis suggested by a genome-wide linkage study. Proc Natl Acad Sci USA 95:10746–10750.

Deighton CM, Roberts DF, Walker DJ. 1992a. Effect of disease severity on rheumatoid arthritis concordance in same sexed siblings. Ann Rheum Dis 51:943–945.

Deighton CM, Gray SW, Biant AJ, Walker DJ. 1992b. Specificity of the *Proteus* antibody response in rheumatoid arthritis. Ann Rheum Dis 51:1206–1207.

Ebringer A, Ptaszynska T, Corbett M, Wilson C, Macafee Y, Avakian H, Baron P, James DCO. 1985. Antibodies to *Proteus* in rheumatoid arthritis. Lancet ii:305–307.

Ebringer A, Cunningham P, Ahmadi K, Wrigglesworth J, Hosseini R, Wilson C. 1992. Sequence similarity between HLA-DR1 and DR4 subtypes associated with rheumatoid arthritis and *Proteus/Serratia* membrane haemolysins. Ann Rheum Dis 51:1245–1246.

Ebringer A, Wilson C, Ahmadi K, Corbett M, Rashid T, Shipley M, et al. 1993. Rheumatoid arthritis as a reactive arthritis to Proteus infection: Prospects for therapy In: Machtey I, ed. "Sixth International Seminar on the Treatment of Rheumatic Diseases". Prog Rheumatol 5, 77–83.

Ebringer A, Rashid T, Wilson C. 2003. Rheumatoid arthritis: Proposal for the use of anti-microbial therapy in early cases. Scand J Rheumatol 32:2–11.

Ebringer A, Hughes L, Rashid T, Wilson C. 2005. Molecular mimicry. In: Vohr HW, editor. Encyclopedic Reference of Immunotoxicology. Berlin Heidelberg: Springer-Verlag. p 451–456.

Fielder M, Tiwana H, Youinou P, Le Goff P, Deonarian R, Wilson C, Ebringer A. 1995. The specificity of the anti-*Proteus* antibody response in tissue-typed rheumatoid arthritis (RA) patients from Brest. Rheumatol Int 15:79–82.

Foxman B, Barlow R, D'Arcy H, Gillespie B, Sobel JD. 2000. Urinary tract infection: Self reported incidence and associated costs. Ann Epidemiol 10:509–515.

Franco AV. 2005. Recurrent urinary tract infections. Best Pract Res Clin Obstet Gynaecol 19:861–873.

Genovese MC, Harris Jr., ED. 2005. Treatment of rheumatoid arthritis. In: Harris Jr, ED, Budd RC, Firestein GS, Genovese MC, Sergent JS, Ruddy S, Sledge CB, editors. Kelley's Textbook of Rheumatology. Philadephia: W.B. Saunders. p. 1079–1100.

Gregersen PK, Silver J, Winchester RJ. 1987. The shared epitope hypothesis. An approach to understanding the molecular genetics of susceptibility to rheumatoid arthritis. Arthritis Rheum 30:1205–1213.

Haraoui B. 2005. Differentiating the efficacy of the tumor necrosis factor inhibitors. Semin Arthritis Rheum 34:7–11.

Ito K, Iida T, Ichino K, Tsunezuka M, Hattori M, Namba T. 1982. Obovatol and obovatal, novel biphenyl ether lignans from the leaves of *Magnolia obovata* Thunb. Chem Pharm Bull 32:3347–3353.

Jawaheer D, Seldin MF, Amos CI, Chen WV, Shigeta R, Monteiro J, Kern M, Criswell LA, Albani S, Nelson JL, et al. 2001. A genome-wide screen in multiplex rheumatoid arthritis families suggests genetic overlap with other autoimmune diseases. Am J Hum Genet 68:929–936.

Kjeldsen-Kragh J, Rashid T, Dybwad A, Sioud M, Haugen M, Forre O, Ebringer A. 1995. Decrease in anti-*Proteus mirabilis* but not anti-*Escherichia coli* antibody levels in rheumatoid arthritis patients treated with fasting and a one year vegetarian diet. Ann Rheum Dis 54:221–224.

Lawrence RC, Helmick CH, Arnett FC, Deyo RA, Felson DT, Giannini EH, Heyse SP, Hirsch R, Hochberg MC, Hunder GG, Liang MH, Pillemer SR, Steen VD, Wolfe F. 1998. Estimates of the prevalence of arthritis and selected musculoskeletal disorders in the United States. Arthritis Rheum 41:778–799.

Lawson AA, Maclean N. 1966. Renal disease and drug therapy in rheumatoid arthritis. Ann Rheum Dis 25:441–449.

Low JM, Moore TL. 2005. A role for the complement system in rheumatoid arthritis. Curr Pharm Des 11:655–670.

MacGregor AJ, Snieder H, Rigby AS, Koskenvuo M, Kaprio J, Aho K, Silman AJ. 2000. Characterizing the quantitative genetic contribution to rheumatoid arthritis using data from twins. Arthritis Rheum 43:30–37.

Merkesdal S, Ruof J, Mittendorf T, Zeidler H. 2004. Cost-effectiveness of TNF-alpha-blocking agents in the treatment of rheumatoid arthritis. Expert Opin Pharmacother 5:1881–1886.

Nasonova VA, Denisov LN, Belen'kii AG, Balabanova RM, Iakovleva DB. 1989. Circulation of intestinal infection in the families of patients with rheumatic diseases and the state of humoral immunity to enterobacterial antigens. Vestn Akad Med Nauk SSSR 6:56–60.

Newkirk MM, Goldbach-Mansky R, Senior BW, Klippel J, Schumacher Jr., HR, El-Gabalawy HS. 2005. Elevated levels of IgM and IgA antibodies to *Proteus mirabilis* and IgM antibodies to *Escherichia coli* are associated with early rheumatoid factor (RF)-positive rheumatoid arthritis. Rheumatology 44:1433–1441.

Newton JL, Harney SM, Wordsworth BP, Brown MA. 2004. A review of the MHC genetics of rheumatoid arthritis. Genes Immun 5:151–157.

Nicolle LE. 1996. Bacteria and urinary tract infections in the elderly. In: Mulholland SG, editor. Antibiotic therapy in urology. Philadelphia: Lippincott-Raven.

Perosa F, Favoino E, Caragnano MA, Prete M, Dammacco F. 2005. CD20: A target antigen for immunotherapy of autoimmune diseases. Autoimmunity Rev 4:526–531.

Rantapaa-Dahlqvist S. 2005. Diagnostic and prognostic significance of autoantibodies in early rheumatoid arthritis. Scand J Rheumatol 34:83–96.

Rashid T, Darlington G, Kjeldsen-Kragh J, Forre O, Collado A, Ebringer A. 1999. *Proteus* IgG antibodies and C-reactive protein in English, Norwegian and Spanish patients with rheumatoid arthritis. Clin Rheumatol 18:190–195.

Rashid T, Tiwana H, Wilson C, Ebringer A. 2001. Rheumatoid arthritis as an autoimmune disease caused by Proteus urinary tract infections: A proposal for a therapeutic protocol. IMAJ 3:675–680.

Rashid T, Leirisalo-Repo M, Tani Y, Hukuda S, Kobayashi S, Wilson C, Bansal S, Ebringer A. 2004. Antibacterial and antipeptide antibodies in Japanese and Finish patients with rheumatoid arthritis. Clin Rheumatol 23:134–141.

Rashid T, Jayakumar KS, Binder A, Ellis S, Cunningham P, Ebringer A, Rheumatoid arthritis patients have elevated antibodies to cross-reactive and non cross-reactive antigens from *Proteus* microbes Ann Rheum Dis (submitted).

Raz R, Chazan B, Dan M. 2004. Cranberry juice and urinary tract infection. Clin Infect Dis 15:1413–1419.

Rogers P, Hassan J, Bresnihan B, Feighery C, Whelan A. 1988. Antibodies to *Proteus* in rheumatoid arthritis. Br J Rheumatol 27(Suppl. 2):90–94.

Senior BW, Anderson GA, Morley KD, Kerr MA. 1999. Evidence that patients with rheumatoid arthritis have asymptomatic 'non-significant' *Proteus mirabilis* bacteriuria more frequently than healthy controls. J Infect 38:99–106.

Stastny P. 1976. Mixed lymphocyte cultures in rheumatoid arthritis. J Clin Invest 57:1148–1157.

Subair H, Tiwana H, Fielder M, Binder A, Cunningham K, Ebringer A, Wilson C, Hudson MJ. 1995. Elevation in anti-*Proteus* antibodies in patients with rheumatoid arthritis from Bermuda and England. J Rheumatol 22:1825–1828.

Tani Y, Tiwana H, Hukuda S, Nishioka J, Fielder M, Wilson C, Bansal S, Ebringer A. 1997. Antibodies to *Klebsiella*, *Proteus*, and HLA-B27 peptides in Japanese patients with ankylosing spondylitis and rheumatoid arthritis. J Rheumatol 24:109–114.

Tishler M, Caspi D, Aimog Y, Segal R, Yaron M. 1992. Increased incidence of urinary tract infection in patients with rheumatoid arthritis and secondary Sjogren's syndrome. Ann Rheum Dis 51:601–606.

Tiwana H, Wilson C, Cunningham P, Binder A, Ebringer A. 1996. Antibodies to four gram-negative bacteria in rheumatoid arthritis which share sequences with the rheumatoid arthritis susceptibility motif. Br J Rheumatol 35:592–594.

Tiwana H, Wilson C, Alvarez A, Abuknesha R, Bansal S, Ebringer A. 1999. Cross-reactivity between the rheumatoid arthritis-associated motif EQKRAA and structurally related sequences found in *Proteus mirabilis*. Infect Immun 67:2769–2775.

Turesson C, Schaid DJ, Weyand CM, Jacobsson LT, Goronzy JJ, Petersson IF, Sturfelt G, Nyhall-Wahlin BM, Truedsson L,

Dechant SA, Matteson EL. 2005. The impact of HLA-DRB1 genes on etra-articular manifestations in rheumatoid arthritis. Arthritis Res Ther 7:1386–1393.

Ushakova MA, Muravijuv UV, Vaneeva NP, Jastrebova NE, Maslov II, Z, acharova IE, et al. 2000. The *Proteus mirabilis* antibody levels in patients with rheumatoid arthritis. Rheumatologia 14: Abst.78.

Wanchu A, Deodhar SD, Sharma M, Gupta V, Bambery P, Sud A. 1997. Elevated levels of anti-*Proteus* antibodies in patients with active rheumatoid arthritis. Indian J Med Res 105:39–42.

Weisbart RH, Min Y, Wong AL, Kang J, Kwunyeun S, Lin A, Kim J, Chan G, Wakelin R, Sohn W, Chang SS, Nishimura RN. 2005. Selective IgA immune unresponsiveness to Proteus mirabilis fumarate reductase A-chain in rheumatoid arthritis. J Rheumatol 32:1208–1212.

Weyand CM, Hicok KC, Conn DL, Goronzy JJ. 1992. The influence of HLA-DRβ1 genes on disease severity in rheumatoid arthritis. Ann Intern Med 117:801–806.

Weyand CM, Seyler TM, Goronzy JJ. 2005. B cells in rheumatoid synovitis. Arthritis Res Ther 7(Suppl. 3):S9–S12.

Wilson C, Corbett M, Ebringer A. 1990. Increased isolation of *Proteus mirabilis* species from rheumatoid arthritis patients compared to osteoarthritis patients and healthy controls. Br J Rheumatol 29(Suppl. II): Abst. 99.

Wilson C, Ebringer A, Ahmadi K, Wrigglesworth J, Tiwana H, Fielder M, Binder A, Ettelaie C, Cunningham P, Joannou C, et al. 1995. Shared amino acid sequences between major histocompatibility complex class II glycoproteins, type XI collagen and *Proteus mirabilis* in rheumatoid arthritis. Ann Rheum Dis 54:216–220.

Wilson C, Thakore D, Isenberg D, Ebringer A. 1997. Correlation between anti-*Proteus* antibodies and isolation rates of *P. mirabilis* in rheumatoid arthritis. Rheumatol Int 16:187–189.

Wilson C, Rashid T, Tiwana H, Beyan H, Hughes L, Bansal S, Ebringer A, Binder A. 2003. Cytotoxicity responses to peptide antigens in rheumatoid arthritis and ankylosing spondylitis. J Rheumatol 30:972–978.

